# Cytokine Storm Combined with Humoral Immune Response Defect in Fatal Hemorrhagic Fever with Renal Syndrome Case, Tatarstan, Russia

**DOI:** 10.3390/v11070601

**Published:** 2019-07-02

**Authors:** Ekaterina Garanina, Ekaterina Martynova, Yuriy Davidyuk, Emmanuel Kabwe, Konstantin Ivanov, Angelina Titova, Maria Markelova, Margarita Zhuravleva, Georgiy Cherepnev, Venera G. Shakirova, Ilseyar Khaertynova, Rachael Tarlinton, Albert Rizvanov, Svetlana Khaiboullina, Sergey Morzunov

**Affiliations:** 1 Openlab “Gene and Cell Technologies”, Kazan Federal University, Kazan 420008, the Republic of Tatarstan, Russia; 2 University Kazan Clinic, Kazan Federal University, Kazan 420008, the Republic of Tatarstan, Russian; 3 Department of Infectious Diseases, Kazan State Medical Academy, Kazan 420012, the Republic of Tatarstan, Russia; 4 School of Veterinary Medicine and Science, University of Nottingham, Sutton Bonington Campus, Loughborough LE12 5RD, UK; 5 Department of Microbiology and Immunology, University of Nevada, Reno, NV 89557, USA; 6 Department of Pathology, University of Nevada, Reno, NV 89557, USA

**Keywords:** HFRS, IL-18, CCL5, SCGF-b, TNF-β, cytokine, fatal case, *Puumala orthohantavirus*

## Abstract

Hemorrhagic fever with renal syndrome (HFRS) is endemic in Tatarstan, where thousands of cases are registered annually. *Puumala* *orthohantavirus* is commonly detected in human case samples as well as in captured bank voles, the rodent hosts. The pathogenesis of HFRS is still not well described, although the cytokine storm hypothesis is largely accepted. In this study, we present a comprehensive analysis of a fatal HFRS case compared with twenty four non-fatal cases where activation of the humoral and cellular immune responses, pro-inflammatory cytokines and disturbed blood coagulation were detected using immunological, histological, genetic and clinical approaches. Multiple organ failure combined with disseminated intravascular coagulation syndrome and acute renal failure was the cause of death. Decreased Interleukin (IL)-7 and increased IL-18, chemokine (C-C motif) ligand (CCL)-5, stem cell growth factor (SCGF)-b and tumor necrosis factor-beta (TNF-β) serum levels were found, supporting the cytokine storm hypothesis of hantavirus pathogenesis.

## 1. Introduction

Hemorrhagic fever with renal syndrome (HFRS) is an acute zoonotic disease endemic in the Republic of Tatarstan, Russia [1]. *Puumala orthohantavirus* (PUUV) is commonly isolated from human HFRS blood and tissue samples as well as bank voles captured in the region [2]. HFRS is characterized by kidney insufficiency and disturbed blood coagulation [3,4,5,6,7]. Acute kidney failure (AKF) and disseminated intravascular coagulation (DIC) syndrome are recognized as the primary causes of death [3,8]. Severe HFRS cases are however rare, with most patients presenting with the milder form of the disease, often referred as nephropathia endemica (NE). NE is characterized by oliguria and thrombocytopenia. The immune reaction to hantavirus infection is believed to be central in HFRS pathogenesis; however, little is known about the mechanisms of hantavirus induced death. This knowledge gap limits the development of effective therapeutics to reduce mortality. 

## 2. Materials and Methods

### 2.1. Subjects

Serum samples from 24 subjects (20 male and 4 female; 44.5 ± 1.8 years) non-fatal and one fatal case (male, 58 years old) admitted to the Agafonov Republican Clinical Hospital for Infectious Disease, Republic of Tatarstan, between Spring 2015 and Fall 2016 were used in the study. Twenty four serum samples were obtained from the early stages (3.6 ± 0.3 days) and eighteen samples from the late stages (10.5 ± 0.8 days) of non-fatal HFRS. Samples from fatal HFRS were collected once, on day seven after admission to the hospital. Diagnosis of HFRS was established based on clinical presentation and was serologically confirmed by detection of anti-hantavirus antibodies. Samples were collected following the standard operation procedure protocol for diagnosis of hantavirus infection. Serum samples from 27 controls without HFRS (21 male and 6 female; 40.8 ± 7.4 year age) were also collected.

### 2.2. Ethics Statement

The Ethics Committee of the Kazan Federal University approved this study, and signed informed consent was obtained from each patient and controls according to the guidelines approved under this protocol (article 20, Federal Law “Protection of Health Right of Citizens of Russian Federation” N323- FZ, 11.21.2011). Informed consent was collected from the next of kin to the fatal HFRS patient.

### 2.3. Animal Samples

Frozen rodent lung tissue samples and information about trapping localities were obtained from the Federal Healthcare Institution “Center for Hygiene and Epidemiology of the Republic of Tatarstan (Tatarstan)”. Rodents were trapped by the village of Vasilevo, the Republic of Tatarstan in May and September 2016. 

### 2.4. Cell Lines and Cell Culture

HEK293FT and Vero E6 cells were obtained from ATCC (American type culture collection, Manassas, VA, USA) and maintained in Dulbecco’s modified Eagle medium (DMEM) supplemented with 10% fetal bovine serum (FBS) (PanEco, Moscow, Russia)), 2 mM L-glutamine, 25 U/mL penicillin, and 25 μg/mL streptomycin (PanEco, Moscow, Russia).

### 2.5. Cloning and Expression of PUUV Glycoproteins

pWPT-PUUM plasmid containing the PUUV glycoprotein coding ORF was purchased from GenScript (Piscataway, NJ, USA). HEK293FT cell monolayers were transfected with the mix of three plasmids: pWPT-PUUMV (vector plasmid), psPAX2 (#12260, Addgene, Watertown, MA, USA) as a packaging plasmid and pCMV-VSV-G (#8454, Addgene, Watertown, MA, USA) as an envelope plasmid. Sixteen hours after transfection, fresh medium was added and supernatant containing the PUUV glycoprotein expressing lentivirus (LV-PuuM) was collected at 98 h. The virus was concentrated by ultracentrifugation (26000 rpm, 4 °C, 2 h) and stored at −80 °C. 

### 2.6. Lentivirus Titer

HEK293FT cells were transduced with serial dilutions of LV-PuuM (10^−1^–10^−9^). Forty eight hours later, cells were trypsinized, washed with PBS (pH 7.4), and incubated with mouse monoclonal anti hantavirus glycoprotein antibodies (1:100 dilution, Abcam, Cambridge, MA, USA) for 2 h at room temperature. Washed (3×, pH 7.4 PBS) cells were incubated with donkey anti-mouse IgG Alexa 647 antibodies (1:1000, Invitrogen, Waltham, MA, USA) for 1 h at room temperature. Washed (3x pH7.4 PBS) cells were fixed (CellFix; BD Biosciences, San Jose, CA, USA) and analyzed for any PUUV glycoproteins expression using BD FACS AriaIII equipment (BD Biosciences, San Jose, CA, USA).

The transduction coefficient (%) is expressed as the ratio of infected cells to the total number of cells in 1 mL. Transduction unit (TU) was calculated as:

TU/mL = number of seeded cells × dilution factor × transduction coefficient (%)/volume of added viral stock.

### 2.7. Hantavirus ELISA

The Hantagnost diagnostic ELISA kit (Institute of Poliomyelitis and Viral Encephalitis, Moscow, Russia) was used to determine the hantavirus-specific antibody titers. 

### 2.8. RNA Extraction and RT-PCR

Total RNA was extracted (100 μL of serum, 50 μg tissue, 10^5^ leukocytes) using TrIzol^®^ reagent (Life Technologies, Carlsbad, CA, USA). cDNA was generated using the Super Script kit (Life Technologies, Carlsbad, CA, USA) according to the manufacturer’s instructions. Single round and nested PCR was used to amplify PUUV products in animal and human samples, respectively. Nested PCR products were sequenced using ABI PRISM 310 and Big Dye Terminator 3.1 Sequencing Kit (ABI, Waltham, MA, USA)) to confirm the hantavirus strain. 

### 2.9. Quantitative PCR (qPCR)

An aliquot of total RNA (40 ng) was used to synthesize the cDNA (Superscript Kit; Invitrogen, Waltham, MA, USA). cDNA (1 μL) was used for the relative quantification of transcripts in a qPCR assay (ThermoFisher, Waltham, MA, USA). Relative values were calculated by normalizing qPCR data to the respective values for glyceraldehyde 3-phosphate dehydrogenase (GAPDH). The fold changes were calculated by ∆∆Ct method by taking the corresponding control group as reference. All qPCR reactions were performed at least in duplicates and repeated three times.. Primer sequences (Evrogen, Moscow, Russia) and expected PCR product sizes are summarized in Table 1. 

### 2.10. DNA Extraction and PCR

DNA was extracted from human blood (100 μL) and cheek swabs using DNA/RNA Extraction Kit (Litekh, Moscow, Russia). The CCR5 genotype was determined by PCR using primers [9] spanning over the exon 4 region of the CCR5 gene. Amplification products were separated by gel electrophoresis.

### 2.11. Multiplex Analysis

Serum levels of 94 analytes were analyzed using Bio-Plex (Bio-Rad, Hercules, CA, USA) multiplex magnetic bead-based antibody detection kits following manufacturer’s instructions. Multiplex kits, Bio Plex Pro Human Cytokine 21-plex and Bio Plex Human Cytokine 27-plex panels were used in the study. Data collected was analyzed using with MasterPlex CT control software and MasterPlex QT analysis software (MiraiBio, Alameda, CA, USA). 

### 2.12. Hantavirus Antibody Epitope Analysis

Hantavirus nucleocapsid and glycoprotein overlapping (15 aa) peptide library was purchased from GeneScript (Piscataway, NJ, USA). Peptide (1 μg/well) was added into the well (96 well plate, HB; Immulon, SPL Lifesciences, Pocheon, Korea) and incubated overnight at 4 °C. Wells were the washed (3×; PBS, 0.1% Tween-20 (PBS-T)), and blocked (2% bovine serum albumin (BSA); Carbonate-Bicarbonate Buffer, pH 9.0) for 1 h at 37 °C and washed again (3 × PBS, 0.1% Tween-20). Serum samples (50 μL) were diluted in PBS-T containing 0.5% BSA, added into the wells in duplicate and incubated overnight at 4 °C. Washed (3× PBS-T), antigen-antibody complexes were incubated with Horseradish Peroxidase (HRP) conjugated goat anti-human IgG antibody (1: 20000; Novex^®^ Cat. No. A24470, (Waltham, MA, USA)) and incubated for 2 hours at 37 °C. At the end of incubation, wells were washed (3× PBS-T) and incubated with 3,3’,5,5’-tetramethylbenzidin (Hema-medica, Moscow, Russia) for 15 min in the dark. Phosphoric acid was added and changes in color were visualized using a spectrophotometer, Tecan Infinite Pro (TECAN, Männedorf, Switzerland).

### 2.13. Neutralizing Antibody Titer

Serial dilutions of serum samples were mixed with lentivirus expressing PUUV glycoproteins (1 × 10^5^ transducing units (TU)/mL) and incubated for 1 h at 37 °C. Vero E6 monolayers (96 well plate; (Corning, Corning, NY, USA)) were inoculated with antibody-antigen mix for 1 h (37 °C, 5% CO_2_), washed with PBS and fresh medium (DMEM, 10% FBS, 2 mM L-glutamine, 25 U/mL penicillin, and 25 μg/mL streptomycin) was added. Five days later cells were fixed and PUUV envelope proteins were probed using mouse anti-Hantavirus glycoprotein G2 antibody (1:100, Abcam, Cambridge, MA, USA) followed by goat anti-mouse Alexafluor 647 (Invitrogen, Waltham, MA, USA). PUUV glycoprotein was detected using a laser confocal microscope LSM800 (Carl Zeiss, Oberkochen, Germany). The fluorescence intensity was analyzed using Image J software (NIH, Bethesda, MD, USA). Neutralizing antibody titer was determined as the reciprocal of the serum dilution which decreased the fluorescence intensity by 50% as compared to that in virus controls (without HFRS serum). A total of 6 fields were counted in each sample.

### 2.14. Leukocyte Population Analysis

Blood sample (200 μL) was collected in ethylenediaminetetracetic acid (EDTA) tubes and used for analysis. Thirteen non-fatal HFRS and twenty seven control blood samples were available for this study. Leukocyte populations were analyzed using anti-human CD3-FITC (cat. 2324020 (Sony Biotechnology, San Jose, CA, USA)), CD4-APC (cat. 2323070 (Sony Biotechnology, San Jose, CA, USA)), CD8-PE (cat. 2322530 (Sony Biotechnology, San Jose, CA, USA)), CD14-Cy7 (cat. 367108 (Biolegend, San Diego, CA, USA)) and CD20-Brilliant Violet421^TM^ antibodies (cat. 302330 (Biolegend, San Diego, CA, USA)). Blood (100 μL) was incubated with anti CD3+CD4+, CD3+CD8+, CD14+ and CD20+, and was analyzed using a BD FACSAria III (BD Biosciences, San Jose, CA, USA). Data was processed with the FlowJo software package (FlowJo LLC, Ashland, OR, USA).

### 2.15. Immunohistochemistry and Immunofluorescent Analysis

Post mortem tissue samples from the lung, kidney, brain, spleen, liver, heart and intestine were collected and fixed in 4% paraformaldehyde for 4 h at 4 °C and cryoprotected with a 30% sucrose solution in PBS. Tissue slides (0.5-μm) were deparaffinized with xylene and rehydrated through a graded alcohol series. Antigen was retrieved using sodium citrate (0.01 M, pH 6.0) at 95 °C for 10 min, rinsed in PBS and incubated in cold methanol for 20 min at –20 °C. Tissue sections were then incubated with serum (matching the host of the secondary antibody) to block non-specific staining (1 h at 37 °C) and incubated with the primary antibody overnight at 4 °C in a humidified chamber. After washing 3 times with PBS-T, the sections were incubated with the secondary antibody for 1 h at 37 °C. Slides were examined using a Zeiss LSM 7000 scanning laser confocal microscope (Carl Zeiss Microscopy, Thornwood, NY, USA) and images were captured with the Zeiss Zen 2009 analysis software.

Tissue morphology was analyzed by light microscopy using eosin-hematoxylin staining.

### 2.16. Phylogenetic Analysis

The nucleotide alignment and phylogenetic analysis of PUUV strains was based on partial S segment sequences of 217 nucleotides in length (nucleotides 378–594). Phylogenetic analysis was done using the MegAlign program (Clustal W algorithm), DNASTAR software package Lasergene v. 7.1.0.44 (DNASTAR, Madison, WI, USA) and MEGA v6.0 [10]. Parameters were adjusted manually. Phylogenetic trees were constructed using Maximum Parsimony method included in Mega v6.0 [10]. 

For phylogenetic analysis we used PUUV partial S segment sequences from the Republic of Tatarstan submitted to the GenBank: PUUV/Laishevo/MG_047/2015, MG573266; PUUV/Teteevo/MG_165/2015, MG573272; PUUV/Vasilyevo/MG_130/2015, MG573296; PUUV/Yash-Ketch/MG_338/2015, MG573279; PUUV/Vysokaya Gora/MG_064/2015, MG573275; PUUV/Yash-Ketch/MG_054/2015, MG573297. Also, several partial S segment sequences of the genetically distinct PUUV strains were downloaded from GenBank database (NCBI) including: Samara_49/CG/2005, AB433843; Puu/Kazan, Z84204; PUUV Udmurtia/894Cg/91, Z21497; CG17/Baskiria-2001, AF442613; Sotkamo 2009, HE801633; PUUV/Konnevesi/Mg_O22B/2005, JQ319168; Kuchuk170/Mg/2007, KJ292966; CRF366, AF367071. Tula virus strain Sennickerode Sen05/205, EU439951 was used as an outgroup.

### 2.17. Statistical Analysis

Statistical analyses were conducted using R language for statistical computing [11], RStudio [12,13] and package “tableone” [9]. Continuous variables were presented with their respective median (M), first (Q1) and third (Q3) quartiles. Categorical variables were presented with cross-tables. Comparisons were carried out using Mann–Whitney test for continuous and Fisher’s exact test for categorical variables. The threshold used for statistical significance was *p* < 0.05.

Statistical analyses were performed using Prism 6.0 software (Graphpad, San Diego, CA, USA) and the p-values were calculated using two-tailed t-tests. Significance was established at a value of *p* < 0.05. Data are presented as mean ± SE. 

## 3. Results

### 3.1. Clinical Summary

#### 3.1.1. Presentation

Patient M, (58 years old, male) from Vasilevo, Tatarstan, was hospitalized with a two day history of weakness, headache and high fever (39 °C) which progressed to vomiting with “coffee grounds” consistency and loss of consciousness. On admission the patient was non-responsive, with pale cyanotic skin and did not have a detectable pulse or arterial blood pressure. The patient had a previous history of hypertension and had been prescribed enalapril (10 mg/day) and aspirin (100 mg/day).

#### 3.1.2. Clinical Testing

Upon admission, hematology analysis revealed leukocytosis, severe thrombocytopenia and decreased prothrombin index (PTI) (Table 2). Coagulopathy was also evident, with high levels of fibrin D dimers and increased activated partial thromboplastin time (aPTT). Increased AST and ALT indicating liver and tissue damage were present on admission. Failing kidney function was also evident with increased creatinine and urea serum levels, as well as protein and red blood cells (RBC) in the urine. Urine output was 700 mL/day. Esophagogastroduodenoscopy (EPGDS) revealed gastric and duodenal bleeding. A CT scan demonstrated subarachnoid bleeding. Antibodies to hantavirus (IgG and IgM) were detected.

#### 3.1.3. Treatment

Multiple transfusions of cryopreserved plasma and thrombocytes were performed increasing thrombocyte count and blood pressure. However, the patient’s condition deteriorated further with progressive multiple organ failure. Oliguria developed with urine output decreased to 50 mL per day. This progressed to anuria by day three after the disease onset.

Due to progressing respiratory failure the patient was intubated and BIPAP (Biphasic Positive Airway Pressure) artificial ventilation was initiated. A medically induced coma was initiated (Atropini 0.1% (0.5 mL) i.v, Promedoli 2% (1.0 i.v); induction with i.v. Natrii oxybutyratis 20% (25.0 mL); myorelaxation with Listenoni 100 mg; continues support with bolus injections of Emulsi Propofol 100 mg). Despite the therapy instituted the thrombocyte count remained low and the patient’s blood pressure further declined. The patient’s cardiac function was supported by an infusion of dopamine (5 µg/kg/min, (350 µg/min); heart sounds were faint. By day 3 the patient’s blood pressure (BP) was 100/65 mmHg, pulse 90 beat/min, temperature 36.7 °C, oxygen saturation was 69%. The patient’s neck muscles were rigid and the skin was pale with multiple hemorrhages. The abdomen was bloated and painful with absent peristalsis. EPGDS at this stage did not show evidence of bleeding. A cerebro-spinal fluid (CSF) sample was taken with analysis demonstrating proteins (2.01 g/L), leukocytosis (39 cells/μL) and a large number of erythrocytes.

**Figure 1:**
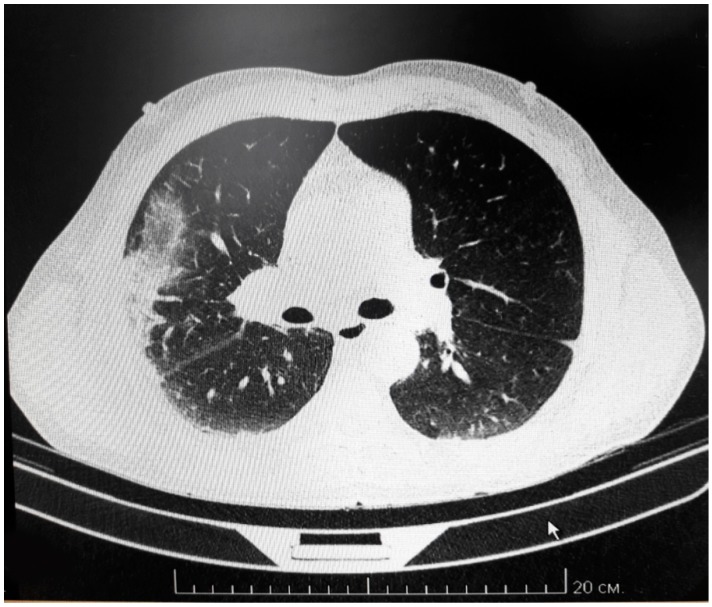
Multi-slice CT of fatal HFRS lung.

The patient’s condition worsened progressively, leading to coma by the evening on day 3 with increasing skin hemorrhages and multiple nasal bleeds that could not be controlled. Hemodialysis was initiated on day 6 with a tracheostomy tube placed for continuing ventilation. Ascites developed on day 11 with 800 mL of fluid drained from the abdomen. By day 13 fibro-bronchoscopy revealed bronchial and pulmonary bleeding. Bleeding was also detected at the site of the peritoneal drainage. Multi-slice computer tomography (CT) revealed multiple ground-glass shadows in the posterior side of both lungs and lateral side of the right lung (Figure 1), indicating pulmonary effusion. Enlarged thoracic lymph nodes were also detected. Sixteen days after admission the patient developed progressive cardio-vascular insufficiency and failure, DIC syndrome, and multi organ failure. Resuscitation was unsuccessful and the patient was pronounced dead.

CT scanning demonstrates multiple small flakes of ground-glass shadows in the back of both lungs. Also, the shadow is found on the lateral side of the right lung. Enlarged lymph nodes are also detected. 

#### 3.1.4. Clinical Diagnosis

Hemorrhagic Fever with Renal Syndrome (HFRS) severe form complicated with acute renal failure (grade 3), DIC syndrome (stage 3), and systemic multiorgan insufficiency (pulmonary, renal and cardiac). Polysegmental pneumonia, severe form (respiratory insufficiency 3 stage, extracorporeal ventilation). Coma stage 1.

A post mortem examination demonstrated hemorrhagic syndrome, pulmonary and tracheobronchial bleeding, blood aspiration, hepatic bleeding. The overall clinical syndrome was consistent with HFRS with acute renal failure, DIC and multiorgan failure. 

### 3.2. Laboratory Analysis (Table 2)

Serial blood biochemistry and hematology analyses using hematology analyzer (Advia 2120i (Simens, Munich, Germany) during the patient’s illness revealed severely impaired kidney function and blood coagulation. Progressively increasing serum levels of creatinine and urea, determined using biochemical analyzer (Architect c8000 (Abbott, Abbott Park, IL, USA) indicate that the function of the kidney was progressively deteriorating. Additionally, the presence of protein and erythrocytes in the urine suggested damage to the glomerulus and filtration membrane of the nephrons. 

Decreased erythrocyte counts and hemoglobin levels (Table 2) were consistent with on-going bleeding. Additionally, drastically decreased thrombocyte counts (Table 2), which were not restored by therapeutic intervention, was considered one of the major causes of the DIC syndrome that developed in this patient. DIC syndrome was also confirmed by increased PTI, fibrinogen level, spontaneous fibrinolysis and the presence of fibrinogen D dimers (Table 2). Despite the intensive care measures, the patient’s blood pressure was not restored. Multiple organ damage was indicated by high serum levels of bilirubin, amylase, ALT, AST, creatinine and urea (Table 2). 

#### 3.2.1. Circulating Leukocyte Analysis

Leukocyte populations, CD3+ CD4+ (CD4 T cells), CD3+ CD8+ (CD8 T cells), CD14+ (monocytes) and CD20+ (B cells), were analyzed in the blood of this fatal HFRS patient and 13 non-fatal HFRS using BD FACSAria III (BD Biosciences, San Jose, CA, USA). Data was processed with FlowJo software package (FlowJo LLC, Ashland, OR, USA) (Table 3). Leukocyte populations did not differ significantly between this fatal and the non-fatal HFRS cases; however, there were markedly higher T cell and B cell counts and lower monocyte counts in the fatal case than the non-fatal cases.

#### 3.2.2. Antibody Response

Serum IgM and IgG antibodies to hantavirus antigens were determined using the Hantagnost commercial diagnostic ELISA according to manufacturer’s instructions (Vektor-best, Novosibirsk, Russia). The titer of hantavirus specific IgM and IgG was 1:600 and 1:400, respectively, suggesting recent hantavirus exposure. Interestingly, IgM and IgG titers were higher in non-fatal HFRS, 1:2260 ± 947.8 and 1:5600 ± 2653.3, respectively. Analysis of hantavirus nucleocapsid antibody epitopes was done using panels of overlapping 15aa peptides (GeneScript, Piscataway, NJ, USA). Interestingly, the serum from the fatal case was lacking in reactivity with any of viral nucleocapsid peptides in contrast to serum from non-fatal HFRS, which reacted with multiple peptides from the PUUV N protein. Since the fatal HFRS serum did react with the whole virus PUUV proteins used in Hantagnost, it could be suggested that the epitopes recognized by this patient are conformational only.

**Figure 2:**
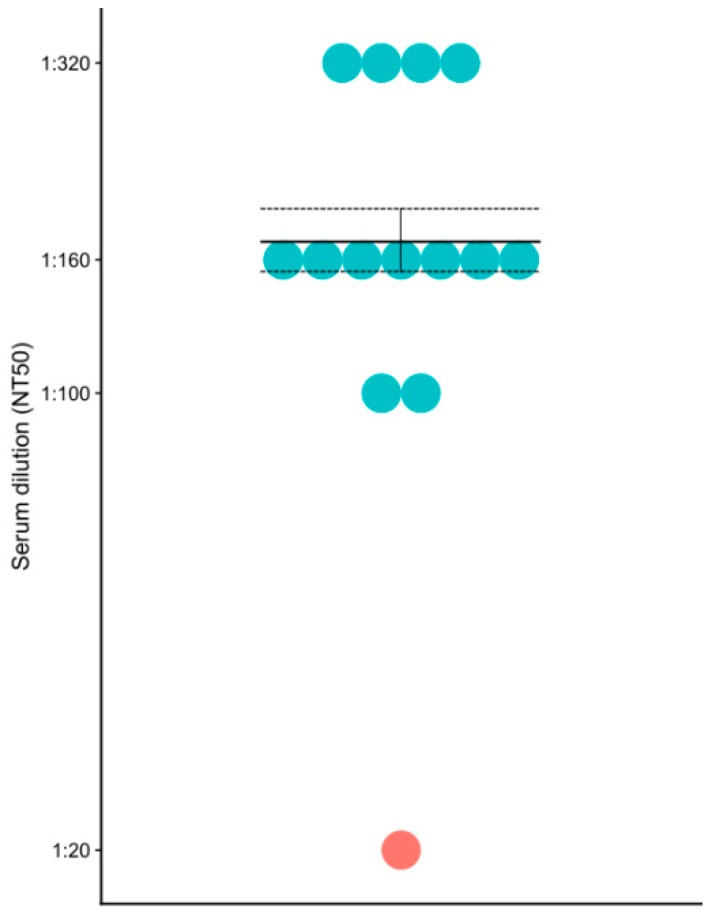
PUUV neutralizing antibodies in the fatal and non-fatal HFRS sera. Blue – non-fatal HFRS; red – fatal HFRS.

Neutralizing antibodies play an important role in protection against hantavirus infection [14]. Therefore, we sought to determine the serum level of PUUV neutralizing antibodies in this fatal case as compared to the non-fatal HFRS cases (Figure 2). Neutralizing antibody titer was determined as the reciprocal of the serum dilution which decreased the fluorescence intensity by 50% as compared to that in the lentivirus control (with no hantavirus protein). A total of 6 fields were counted in each sample. Neutralizing antibody titer in the non-fatal HFRS cases varied from 100 to 320 however, the titer of neutralizing antibodies in the fatal HFRS was 20, which was much lower than that of the non-fatal cases. 

The defect in humoral response was evident in this fatal HFRS case. Similar data was presented by McNeil et al., demonstrating lack of hantavirus specific IgG in many fatal hantavirus pulmonary syndrome (HPS) cases, while all non-fatal cases had detectable level of antibodies [15] Also, a lower titer of neutralizing antibodies in severe hantavirus infection was demonstrated by Bharadwaj et al. [16]. In another report, low anti-PUUV antibodies were shown to be associated with severe forms of infection [17]. These authors suggested that passive immunotherapy could be beneficial for severe PUUV infection. This assumption is also supported by our observation, demonstrating a lack of hantavirus specific antibodies in fatal HFRS. 

#### 3.2.3. PUUV RNA

**Figure 3:**
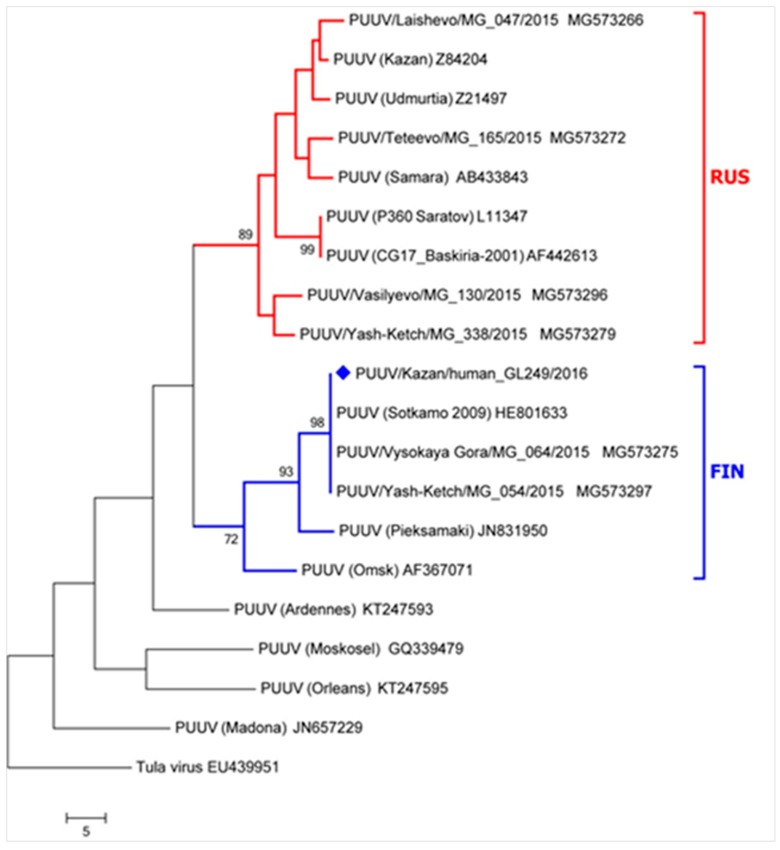
Phylogenetic analysis of PUUV RNA from fatal HFRS case. The sequence of the fatal case is marked by a blue diamond. The nucleotide alignment and phylogenetic analysis of PUUV strains was based on partial S segment sequences of 217 nucleotides in length (nucleotides 378–594). The tree branch lengths were calculated using the average pathway method and have a scale representing the number of changes in the sequence. Red – RUS lineage; Blue – FIN lineage.

A PUUV strain (Figure 3), clustering together with isolates from different regions of Russia, was detected in the blood sample from the fatal case. Phylogenetic analysis revealed that this PUUV was closely related to the strains isolated from Finland (Finnish group). 

#### 3.2.4. Immunohistochemistry and Immunofluorescent Analysis

**Figure 4:**
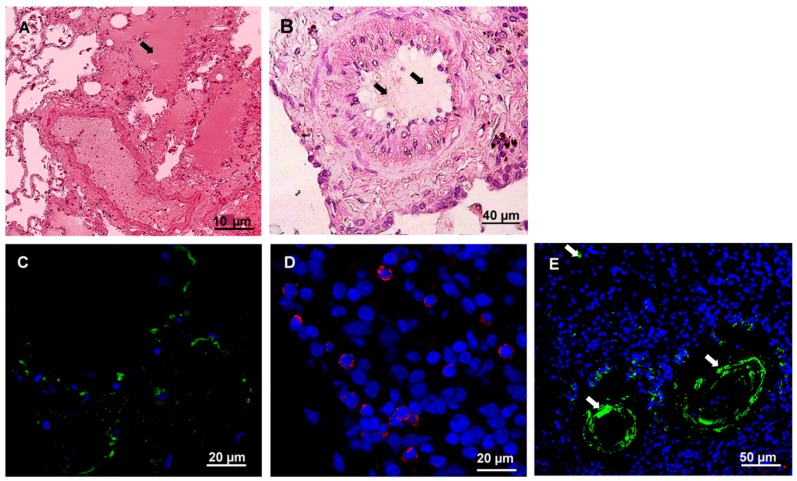
Histochemistry and immunofluorescence analysis of the lung tissue from fatal HFRS. Lung tissues (0.5–3-μm thick) were deparaffinized before histochemistry and immunofluorescence analysis. **A** and **B**. Tissue samples were stained with hematoxylin and eosin (H&E). Arrow points at exudate in the lung alveolus and bronchiole. **C**. hantavirus nucleocapsid protein (1:100, Santa Cruz Biotechnology, Dallas, TX, USA) and anti-mouse Alexa 488 (1:1000; Invitrogen, Waltham, MA, USA); **D**. CD4+ leukocytes (polyclonal anti-CD4 (1:100, Abcam, Cambridge, MA, USA) and anti-rabbit Alexa647 (1:1000; Invitrogen, Waltham, MA, USA); **E**. CD20+ leukocytes (monoclonal anti-CD20 (1:50, Abcam, Cambridge, MA, USA) and anti-mouse Alexa 488 (1:1000; Invitrogen, Waltham, MA, USA); DAPI – used to visualize nucleus. White arrows show positive CD20+ infiltrating leukocytes.

**Figure 5:**
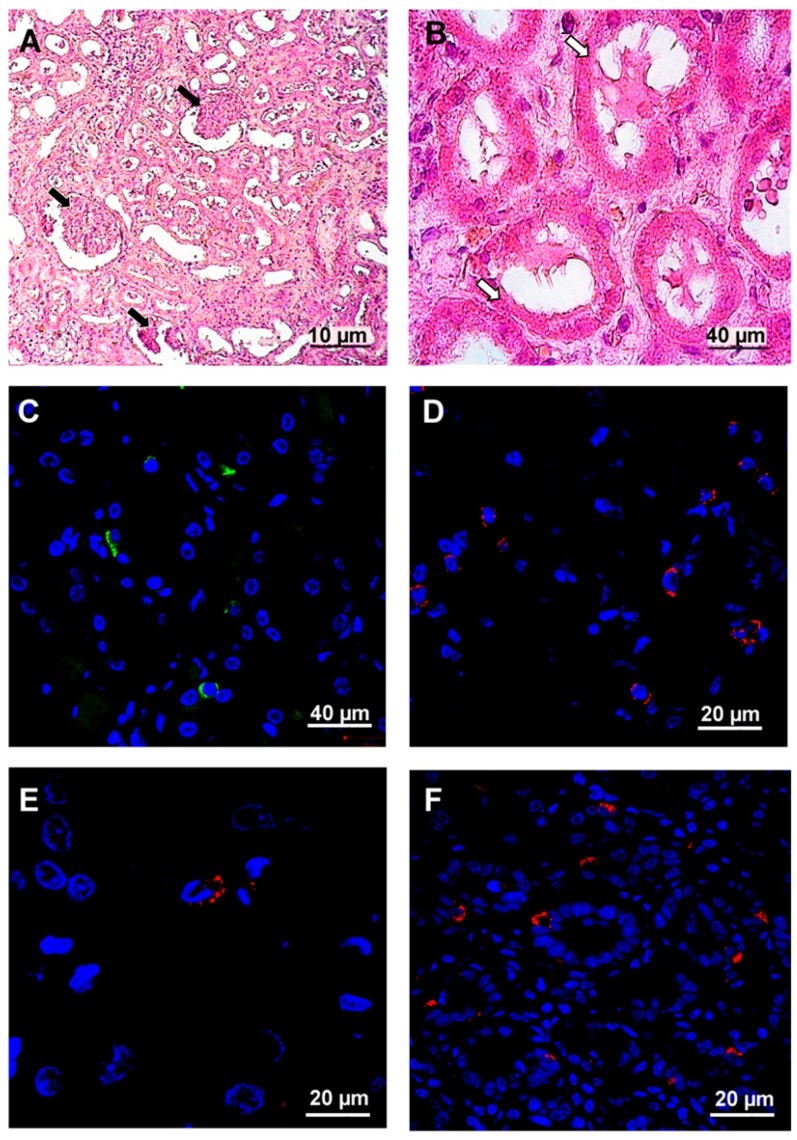
Histochemistry and immunofluorescence analysis of kidney from fatal HFRS. Kidney tissues (0.5–3-μm thick) were deparaffinized before histochemistry and immunofluorescence analysis. **A** and **B**. Tissue samples were stained with hematoxylin and eosin (H&E). Arrows in the figure show whit changes in kidney structure. White arrows—kidney glomeruli exhibited wrinkling epithelium. Black arrows mark the plasmorrhexis of kidney. **C**. hantavirus nucleocapsid protein (1:100, Santa Cruz Biotechnology, Dallas, TX, USA) and anti-mouse Alexa 488 (1:1000; Invitrogen, Waltham, MA, USA). **D**. CD4+ (1:100, Abcam, UK) and anti-rabbit Alexa 647 (1:1000; Invitrogen, Waltham, MA, USA). **E**. CD8+ (1:100, Abcam, UK) and anti-mouse Alexa 647 (1:1000; Invitrogen, Waltham, MA, USA) **F**. clusterin (1:100, Santa Cruz Biotechnology, Dallas, TX, USA) and anti-mouse Alexa 647 (1:1000; Invitrogen, Waltham, MA, USA). DAPI was used to visualize the nucleus.

Post mortem tissue samples from the lung, kidney, brain, spleen, liver, heart and intestine were collected and used for histology and immunohistochemistry analysis. The most significant changes in tissue structures and leukocyte infiltration were detected in the lungs (Figure 4) and kidney (Figure 5).

The lung structures were severely damaged, and destruction of the alveoli was detected (Figure 4A). Also, exudate was found in the alveoli as well as thickened alveoli walls. Hemostasis was evident in the blood vessels. Additionally, mucus and blood were detected inside of bronchioles (Figure 4B). These findings are consistent with the diagnosis of pneumonia based on spiral computer tomography findings. Immunofluorescent analysis demonstrated hantavirus antigens in the lung (Figure 4C). Moderate leukocyte infiltration containing CD4+ and CD20+ lymphocytes, is detected (Figure 4D and E). 

Glomerular and tubular damage was detected in kidney tissue (Figure 5A,B). Kidney glomeruli were shrunk which could be explained by the previous history of long lasting hypertension (Figure 5A). Additionally, tubular epithelial cells were flattened with plasmorrhexis (Figure 5B). Some cells presented with karyorrhexis. Hantavirus antigens were detected in tubular epithelial cells (Figure 5C). CD4+, CD8+ and CD20+ lymphocyte infiltration was detected in kidney tissue (Figure 5D, E and F). Also, clusterin expression (which has been previously associated with HFRS) [1,18] was demonstrated at the basal side of the tubular epithelial cells (Figure 5G). Immunofluorescent analysis revealed that infiltrating leukocytes were CD4+, CD8+ and CD20+, suggesting that lymphocytes (T-helpers, cytotoxic T cells (CTL) and B cells) are primarily migrating across the endothelium.

PUUV RNA was detected in circulating CD8+ and CD14+ leukocytes using qPCR. In contrast, viral genome was not detected in CD4+ and CD20+ lymphocytes (Table 4).

Leukocyte populations were separated using BD FACSAria III and used for RNA extraction (purity of leukocyte populations was 94.3% ±2.6%). PUUV RNA was detected by qPCR. Relative values were calculated by normalizing qPCR data to the respective GAPDH. The fold changes were calculated by ∆∆Ct method by taking the corresponding control group as reference. Data are presented as mean ± SE. Significance was established at a value of *p* < 0.05. 

#### 3.2.5. Serum Cytokines

Serum from the terminal case was collected on the 7th day after hospitalization and used to identify cytokines linked to the HFRS lethal outcome (Table 5). Analytes in serum from 24 early (3.6 ± 0.3 days) and 18 late (10.5 ± 0.8 days) cases of non-fatal HFRS were compared to those in the fatal case. First, cytokines for which the level in the fatal case was outside the range found in early and late HFRS were identified. Next, the coefficient of difference (CD) was determined by dividing the cytokine value in the fatal case by the value of the 99% confidence interval for the same cytokine in non-fatal early or late HFRS patients. A CD higher or lower than 2.0 was considered as significant. 

The level of the majority of cytokines was within the range detected in the early and late HFRS sera. However, levels of several cytokines (IL-7, IL-18, CCL5, SCGF-b and TNF-β) differed between fatal and non-fatal HFRS. The level of IL-7 was at the lowest in the early non-fatal HFRS. When compared to non-fatal late HFRS, level of IL-7 was 11.65 times lower in the fatal HFRS case. Fatal case serum was collected on day 7 of the disease, which is close to the average time of serum collection in the late stage HFRS patients (10.5 ± 0.8 days). Therefore, it could be expected that IL-7 levels should be increasing in the fatal HFRS. Instead, the interleukin levels remained low, suggesting a deficiency in IL-7 pathways. IL-7 is produced by the thymus, bone marrow and lymph node stroma cells [19,20]. The absence of IL-7 signaling is responsible for a failure to develop γδT cells and NK-T cells [21,22], which are essential for anti-viral protection [23,24]. Also, IL-7 promotes survival of NK cells by inhibiting their apoptosis [25]. Interestingly, activation and proliferation of NK cells has been demonstrated in HFRS and HPS [26,27], strongly indicating the role of these leukocytes in pathogenesis of hantavirus infection. Our data further support the importance of NK in HFRS pathogenesis, and more so a potential protective role of these cells.

In contrast to IL-7, levels of IL-18, CCL5, SCGF-b and TNF-β were very much higher in the fatal cases serum as compared to early and late HFRS cases. These cytokines have pleiotropic effects linked to stem cell proliferation, mononuclear leukocyte chemotaxis and inflammation; however, one shared feature is that these cytokines target mononuclear leukocytes. SCGF-b triggers proliferative activity of hematopoietic progenitor cells [28] and, together with Flt-3 Ligand and VEGF, it can promote the CD34 + CD133+ progenitors expansion and differentiation into the endothelial cells [29,30]. TNF-β plays a central role in establishing lymphoid microenvironments, host defense and inflammation. Just like SCGF-b, TNF-β has been implicated in pathogenesis of inflammation [31,32]. One way TNF-β is thought to contribute to inflammation by inducing the secretion of CCL5 [33]. CCL5 is thought to contribute to inflammation by increasing chemotaxis of activated mononuclear leukocytes [34,35,36]. Transendothelial migration of leukocytes is regulated by interaction between CCL5 and its receptor CCR5 [37], where the expression of the wild type CCR5 receptor is essential for the effective chemotaxis [38,39]. Interestingly, the wild type CCR5 genotype was identified in the fatal HFRS case, suggesting the most effective interaction between the ligand (CCL5) and the receptor. 

The role of inflammation in pathogenesis of fatal HFRS is supported by finding a high level of IL-18 in the serum of the fatal case. IL-18 has been linked to a severe inflammation [40] and was suggested as a predictor for a severe outcome [41,42] in inflammatory diseases. We have previously shown the importance of IL-18 in pathogenesis of fatal hantavirus infections [27], where increased cytokine levels were detected in fatal hantavirus pulmonary syndrome (HPS) cases. IL-18 could contribute to inflammation by upregulation of vascular cell adhesion molecule-1 (VCAM-1) and E-selectin expression in endothelial cells [43]. In addition to inflammation and leukocyte chemotaxis, IL-18 together with IL-15 and IL-12 induces a Th1 immune response [44]. Therefore, it could be suggested that the massive migration of mononuclear leukocytes contributes to pathogenesis of the fatal HFRS.

Massive failure of blood tissue barriers was evident in this fatal HFRS case as bleeding and ascites were continuously reported in this patient. The involvement of the gastro-intestinal tract (GIT), especially in having a compromised blood-tissue barrier at the earliest stages of the disease, was supported by the clinical finding of “coffee grounds” vomiting and GI bleeding.

### 3.3. Local Hantavirus Epidemiology

Lung samples from seven small rodents captured around Vasilievo village were collected and used for RNA extraction. Two out of seven (28.6%) bank voles were positive for PUUV RNA, which was later identified as RUS genetic lineage (strain PUUV/Vasilevo/MG_130/2015; Figure 3). Interestingly, the PUUV from fatal HFRS was identified as FIN genetic lineage (Figure 3), which has been previously found in non-fatal HFRS cases from Republic of Tatarstan [45]). Therefore, we suggest that the fatal outcome in this HFRS case could be related to the hosts’ reaction to infection rather than the viral strain. PUUV strains containing the S-segment of the RUS and FIN lineages were also found in the bank vole populations in the areas adjacent to Vasilievo. Some of these strains were included into the phylogenetic analysis in this study (Figure 3). The nucleotide sequence of these strains was 98%–100% identity to the sequences detected in the non-fatal HFRS patients. Thus, similar PUUV strains were identified in captured bank voles and in HFRS patients. 

## 4. Conclusions

PUUV remains an emergent infection in Republic of Tatarstan, Russia. HFRS is an acute zoonotic infection that is caused by PUUV. The HFRS incidence rate in Republic of Tatarstan is one of the highest in Russia, with over 1000 cases being registered annually [1,46]. The disease is normally mild, with complete recovery of the majority of patients. However, fatal HFRS have been documented during some outbreaks, with a mortality rate of 0.43% [1]. Cytokine storm and excessive immune response have been suggested to play a central role in pathogenesis of hantavirus infection [47,48,49,50]. Our data further confirms this hypothesis, where increased cytokine and chemokine production was demonstrated in this fatal HFRS case as compared to non-fatal cases. It should be noted that the level of IL-18 was significantly higher in fatal HFRS than in non-fatal. This cytokine was implicated into pathogenesis of the severe form of the disease and fatal outcomes of cardiovascular diseases, sepsis, allergic reactions, and ulcerative colitis [41,44,51,52,53]. Also, we have shown that IL-18 was significantly upregulated in fatal HPS cases [27]. Therefore, we propose that IL-18 is important for pathogenesis of cytokine storm, with high likelihood of a fatal outcome in an HFRS case. 

We propose that differences in a patient’s humoral immune response to the virus could be a contributing factor in fatal HFRS. Differences in epitope recognition by antibodies from patients with different outcomes of the disease are reported regularly [54,55,56]. It has been suggested that changes in epitope recognition could lead to inability of antibodies to interfere with virus replication [1,54]. In the present HFRS case, changes in the epitope recognition were associated with a low neutralizing antibody titer, which could contribute to pathogenesis and the fatal outcome.

Taken together, our data confirms the cytokine storm hypothesis of hantavirus pathogenesis, presenting IL-18 as its potential leading force. Significant changes in hantavirus epitope recognition and weak neutralizing antibody production could also contribute to failure of immune protection against PUUV. Cytokine driven leukocyte infiltration, including CTL, could cause a detrimental effect on tissue structure, thereby promoting vascular leakage and further contributing to pathology in HFRS.

## Figures and Tables

**Table 1:** Primers used in the study.

Name of the Primer	Nucleotide Sequence	PCR Product Size
RT-PCR		
PuuV-For 1st round	5′-CTGCAAGCCAGGCAACAAACAGTGTCAGCA-3′	723 bp
PuuV-Rev 1st round	5′-GTCTGCCACATGATTTTTGTCAAGCACATC-3′	
PuuV-For 2st round	5’-AAGTGGCCAGACAGCAGATT-3’	217 bp
PuuV-Rev 2st round	5’-GGCAGTAGGCATGGAAACAT-3’	
qPCR		
PuuVN-F	5′-AGGCAACAAACAGTGTCAGC-3′	186 bp
PuuVN-R	5′-AATCCCAGTCGGGTCAGTAG-3′	
GAPDH-F	5′-TTTGGCTACAGCAACAGG-3′	107 bp
GAPDH-R	5′-GGTCTCTCTCTTCCTCTTG-3′	

**Table 2:** Clinical laboratory data dynamics.

Day-Post Admission		0	2	4	7	9	11	12	14	15
	**Normal Values**									
AP (mm/Hg)		90/50	100/60	120/80	115/70	110/70	114/70	100/75	90/60	
**Blood test**										
Leukocyte (10^9^ cell/L)	3.5–11.0	14.74	18.48	13.6	16.67	8.9	7.3		4.9	4.5
Hemoglobin (g/L)	130–170	140	137	95	103	101	82		78	91
Erythrocyte (10^12^ cell/L)	4.2–6.9	4.91	4.87	3.2	3.42	3.49	2.95		2.79	3.2
Hematocrit	39–62	39.6	38.4	27	29.13	31	25.7		24.3	28.3
Thrombocyte (10^9^ cells/L)	140–450	5	3	39	65	21	49		38	53
**Arterial Blood Gas test**										
pH	7.45		7.07	7.47	7.44	7.43		7.4		7.2
P CO_2_ (mmHg)	33–45		24.5	28.9	35.4	40.6		32.3		52.1
P O_2_ (mmHg)	75–100		45.5		46.6	41.8		41.2		65.4
HCO_3_^-^ (mEq/L)	21–28		13.3	20,6	23.6	29.1				24.8
**BE**			-8.8	-2.6	-1			1,6		-2.4
0_2_ SAT (%)	94–100		74.7					81		89
**Biochemical analysis**										
K+ (mM/L)	3.5–5.0		4.9	4.0	4.3	4.3	6.6	5.4	5.1	7.3
Na+ (mM/L)	135–145		141	143	146	141	140	144	142	141
Cl- (mM/L)	95–105		92	95	99	96		90		95
Bilirubin (μM/L)	2–25	20	47.1			49	54.9			
Bilirubin, direct (μM/L)	0–9	9	7							
PTI (%)	72–123	55	69	60	78					
PTI by Quick (%)	78–142			49.8					61	66.3
Fibrinogen B (mg/dL)	1.7–4.2	1.9		2.5	1.8	3.3		6		
Spontaneous fibrinolysis (%)	10–20			˃50	˃40	˃45	˃40	˃40	˃40	˃40
Soluble fibrinomonomeric complexes (SFMC) (U)	0.36–0.48			5.5		4.5	6.5	4		
Activated partial thromboplastin time (aPTT) (s)	30–40	51	29	38.2		43	40	39.8		
Antithrombin (AT) lll (%)	76–126						86.6%	56%		
International normalised ratio (INR)	0.82–1.18	1.7	1.54	1.69	1.2	1.2		1.2	1.46	1.37
D dimer (ng/μL)		+ + ++	+ + +							
Procalcitonin (ng/mL)	0–0.15					12.8	12.8	˃12.8		
C-reactive protein (CRP) (mg/L)	0–6			48				96		
Amylase (U/L)	25–190	140			600	852				
Alanine aminotransferase (ALT) (U/L)	5–56	510	1211	1250	389	98		21		
Aspartate transaminase (AST) (U/L)	8–40	738	2316	2400	500	136		40		
Lactate dehydrogenase (LDH) (U/L)	140–280			3328						
Creatinine (μM/L)	60–118	286	547				949			
Urea (mM/L)	3–7	14	19.9	43	29	50	65	56		
**Urinalysis**										
Protein (g/L)	absent	0.15	0.9	4		4				1.54
Red Blood Cells (RBC) (cells/field)	0–3	10–20	4–5	many		many				many

**Table 3:** Circulating leukocyte populations in fatal and non-fatal HFRS.

	CD3+CD4+ (%)	CD3+CD8+ (%)	CD14+ (%)	CD20+ (%)
Fatal	6	11	0.7	5.2
Non-fatal	3.13 ± 3.78	6.05 ± 4.12	3.03 ± 1.66	3.33 ± 1.57

Data are presented as mean ± SE. Significance was established at a value of *p* < 0.05.

**Table 4:** PUUV RNA detection in various leukocyte populations.

Leukocyte Populations	Fold Changes, *p* < 0.05
CD4+	0.94 ± 0.02
CD8+	28.98 ± 0.02
CD14+	25.11 ± 2.0
CD20+	0.86 ± 0.11

**Table 5:** Serum urea, creatinine and cytokine levels in fatal and non-fatal (early and late) HFRS.

Analyte	Fatal (pg/mL)	Late HFRS		Early HFRS	
		pg/mL	Fold Change	pg/mL	Fold Change
**Urea**	14.00	4.00 [2.50; 6.3]	**2.22**	7.00 [0; 38.00]	
Creatinine	286.00	112.00 [77.00; 142]	**2.01**	131.00 [18.00; 545.00]	
Interferon alpha-2 (IFN-2a)	53.95	19.50 [10.90; 125.03]		21.24 [2.24; 129.35]	
Interferon gamma (IFN-γ)	41.69	6.26 [3.38; 95.52]	1.25	57.78 [5.73; 1026.17]	1.25
IL-1a	6.26	0.14 [0.02; 5.00]		0.14 [0.00; 5.00]	
IL-1b	5.67	2.52 [0.99; 149.36]		2.10 [0.22; 308.78]	
IL-1ra	149.90	91.69 [3.85; 1489.14]		76.92 [3.72; 3175.21]	
IL-2Ra	195.57	36.76 [3.00; 4745.66]	0.04	84.29 [3.00; 4745.66]	
IL-2	10.98	7.25 [2.50; 33.40]		5.57 [0.25; 241.84]	
IL-3	203.16	59.27 [5.00; 376.26]		88.18 [5.00; 2806.74]	
IL-4	26.58	4.45 [1.45; 14.37]	1.85	3.38 [0.02; 29.77]	0.89
IL-5	4.05	5.10 [0.43; 59.73]		4.64 [0.19; 29.57]	
IL-6	18.73	16.45 [1.04; 15099.02]		9.82 [0.83; 15099.02]	
IL-7	0.20	9.05 [2.33; 26.61]	**−11.65**	5.75 [0.18; 96.86]	
IL-8	95.57	32.00 [11.00; 266.19]		44.00 [3.00; 1058.80]	
IL-9	9.43	37.49 [4.62; 318.27]		33.33 [1.36; 213.48]	
IL-10	31.62	25.37 [8.61; 460.05]		17.48 [1.73; 832.71]	
IL-12(p40)	1045.95	78.73 [9.00; 1207.96]	0.87	165.15 [1.70; 4220.54]	0.25
IL-13	21.51	4.77 [0.83; 14.76]	1.46	4.57 [0.02; 68.04]	
IL-15	81.59	21.51 [3.52; 54.74]	1.49	16.38 [1.20; 143.74]	0.57
IL-16	898.02	151.19 [5.00; 658.51]	1.36	174.41 [5.00; 3330.59]	0.27
IL-17	79.91	21.13 [2.05; 91.25]		9.95 [0.53; 220.06]	
IL-18	351.23	11.23 [0.99; 99.78]	**3.52**	14.53 [0.07; 137.16]	**2.56**
IL-22	17.52	6.80 [4.80; 45.00]		6.00 [2.00; 65.00]	
CCL2	104.89	33.25 [3.91; 310.57]		30.00 [3.27; 2958.67]	
CCL3	4.34	3.12 [0.56; 51.98]		2.15 [0.09; 51.98]	
CCL4	111.92	70.88 [13.58; 897.03]		46.62 [4.50; 897.03]	
CCL5	21833.09	133.00 [12.00; 7120.00]	**3.07**	124.98 [1.02; 8354.01]	**2.61**
CCL7	49.54	8.30 [1.35; 45.77]	1.08	13.56 [0.87; 327.46]	
CCL11	65.05	69.12 [6.27; 496.41]		39.73 [2.78; 666.70]	
CCL27	155.80	45.37 [4.00; 200.06]	0.78	47.10 [0.04; 200.06]	0.78
Chemokine (C-X-C motif) ligand (CXCL)1	214.63	26.11 [7.00; 123.68]	1.74	22.90 [1.50; 77.35]	**2.77**
CXCL9	5199.74	1087.58 [2.00; 6951.33]	0.75	1683.49 [2.00; 38365.63]	
CXCL10	4301.79	675.40 [22.07; 14148.17]	0.30	508.88 [16.86; 14685.22]	
Basic fibroblast growth factor (FGF basic)	18.02	15.74 [0.67; 73.25]		8.96 [0.56; 283.31]	
Granulocyte-colony stimulating factor (G-CSF)	36.41	25.33 [8.85; 235.96]		24.20 [0.87; 2358.95]	
Granulocyte- macrophage colony-stimulating factor (GM-CSF)	16.73	11.42 [1.20; 85.95]		5.04 [0.21;704.31]	
Leukemia inhibitory factor (LIF)	17.89	11.00 [1.20; 15.73]	1.14	5.28 [0.32; 144.47]	0.12
Macrophage colony-stimulating factor (M-CSF)	41.56	7.00 [0.68; 9.6]	**4.33**	5.69 [0.31; 32.25]	1.29
Macrophage migration inhibitory factor (MIF)	274.66	111.42 [0.42; 1440.27]		162.41 [6.00; 1951.05]	
Hepatocyte growth factor (HGF)	1153.94	40.45 [1.59; 707.00]	1.63	109.26 [3.23; 4732.69]	0.24
Nerve growth factor b-(NGF)	7.32	4.00 [0.72; 5.10]	1.44	4.00 [0.32; 16.44]	0.45
Stem cell factor (SCF)	186.95	38.60 [7.00; 95.30]	1.96	48.99 [7.00; 566.30]	0.33
SCGF-b	115197.14	4131.02 [39.00; 14076.65]	**8.18**	5740.14 [39.00; 50992.29]	**2.26**
TNF-β	54.64	0.31 [0.09; 5.00]	**10.93**	0.40 [0.14; 3.00]	**18.21**
Tumor necrosis factor related apoptosis-inducing ligand (TRAIL)	42.91	12.12 [0.24; 225.08]		27.54 [0.46; 225.08]	
Tumor necrosis factor (TNF)-α	14.39	35.91 [2.23; 194.11]		23.63 [1.44; 2117.67]	
Platelet-derived growth factor (PDGF)-bb	129.28	267.95 [32.09; 3903.78]		414.96 [3.00; 4291.09]	
Vascular endothelial growth factor (VEGF)	20.38	99.26 [1.72; 1822.04]		67.12 [1.27; 1822.04]	

Statistical analyses were conducted using R language for statistical computing [11], RStudio [12,13] and package “tableone” [9]. Continuous variables were presented with their respective median (M), first (Q1) and third (Q3) quartiles. Categorical variables were presented with cross-tables. Comparisons were carried out using Mann–Whitney test for continuous and Fisher’s exact test for categorical variables. The threshold used for statistical significance was *p* < 0.05. Red – significantly increased; Blue – significantly decreased.
